# Structural and Functional Properties of Underutilised Cowpea and Moth Bean Starches

**DOI:** 10.3390/foods14213647

**Published:** 2025-10-26

**Authors:** Weiyan Xiong, Minqian Zhu, Surya P. Bhattarai, Sushil Dhital

**Affiliations:** 1 Department of Chemical and Biological Engineering, Monash University, Clayton Campus, Melbourne, VIC 3800, Australia; weiyan.xiong@monash.edu (W.X.); minqian.zhu@monash.edu (M.Z.); 2 Institute for Future Farming Systems, CQUniversity, Australia, Rockhampton, QLD 4701, Australia; s.bhattarai@cqu.edu.au; 3 Centre for Crop Health, University of Southern Queensland, Toowoomba, QLD 4350, Australia

**Keywords:** starch, cowpea, moth bean, physicochemical properties

## Abstract

Starches isolated as by-products from protein extraction of three cowpea and three moth bean cultivars were investigated for their structural and functional properties, including particle size, apparent amylose content (AAC), crystallinity, gelatinisation and retrogradation behaviour, pasting properties, and gel texture. Cowpea starches exhibited higher AAC, gelatinisation temperatures, retrogradation enthalpy, and gel strength, indicating greater thermal stability and stronger gel network formation. In contrast, moth bean starches showed lower ACC, higher relative crystallinity, and greater gelatinisation enthalpy, reflecting more compact native crystalline structures, due to a higher amylopectin content. The lower AAC of moth beans resulted in limited retrogradation and softer gels. To evaluate the multivariate relationships among the starch samples, cluster analysis was performed, which grouped the samples according to botanical origin. This classification underscored the distinct structural and functional attributes differentiating cowpea and moth bean starches. These findings provide insight into cultivar-dependent starch behaviour. Cowpea starches may be suited for applications requiring thermal stability and a firm texture, such as noodle formulations and microwave foods, while moth bean starches offer potential for products with smooth textures and low retrogradation, such as in instant or ready-to-eat food products.

## 1. Introduction

Cowpea (*Vigna unguiculata* L.) is a versatile tropical legume, grown worldwide in 14.5 million hectares, with a total annual production of 6.5 million metric tonnes, with Africa accounting for over 80% of cowpea production [1,2]. Despite its growing importance in food, feed, and plant protein extraction markets, cowpea production remains limited; it is primarily grown as green manure crops and there is only small-scale production for seed and feed. One of the major constraints is the lack of cultivars with heat and drought tolerance, as well as limited exploration of high-value food applications [3]. In this context, moth bean (*Vigna aconitifolia* L.) is a more drought and heat-tolerant legume option [4], and hence offers potential for incorporating it into broad-acre farming systems in dry tropical regions of Australia. Despite its greater agro-ecological adaptability for the tropics, moth bean still remains a relatively minor crop compared to major legumes, and is underutilised in global food systems [4,5].

Vigna seeds are rich in both protein and starch, with protein content typically ranging from 20 to 30% and starch content ranging from 45 to 60% on a dry-weight basis, depending on variety and growing environments. Vigna protein is a good source of essential amino acids, while the carbohydrates are largely composed of starch, making it a nutrient-dense food. Starch is a major component of all grain legumes, including Vigna [6], which hence plays a significant role in its functional and nutritional properties [7,8]. In particular, starches from cowpeas and moth beans offer promising functional properties that are increasingly valued in both food and industrial applications. Compared to conventional starches such as maize and potato, cowpea starch tends to have a higher apparent amylose content, which is beneficial for gel strength and retrogradation [9], while moth bean starch demonstrates high viscosity and superior film-forming ability, making it suitable for biodegradable films and viscous food formulations [10]. As the demand grows for more diverse and sustainable starch sources, underutilised legumes are drawing increasing interest, due to their value for tolerance to harsh environmental conditions and potential to serve as alternative starch sources in both food and industrial applications. Among these, cowpeas and moth beans represent promising candidates, particularly for tropical and semi-arid regions [11,12], yet their starch characteristics remain largely underexplored compared to major starch crops such as maize, potato, and rice [13].

Significant genetic variation exists in germplasm for cowpea and moth bean’s seed quality attributes and adaptation to tropical conditions [14,15]. Furthermore, crop genetics have been shown to influence starch structure and properties, such as granule morphology, particle size, amylose and amylopectin ratio, crystalline structure, and gelatinisation behaviour [16,17]. However, few studies have systematically compared the starch properties of legumes while considering both differences between species (cowpea vs. moth bean) and variations among cultivars within the same species. This gap hinders our ability to differentiate the relative contributions of species-level and cultivar-level variation from functional starch traits. In light of these limitations, in this study, there are two contrasting Vigna species (cowpea and moth bean), each with three cultivars, and each representing genetic diversity with tropical adaptation potentials. All samples were grown under uniform agronomic practices, and starch was extracted from mature seeds. Comprehensive characterisation of starch properties was conducted to provide a detailed comparative analysis of the structural and functional properties of starch across and within the two underutilised Vigna species. By elucidating the physicochemical diversity of these starches, the study contributes to a broader understanding of their potential application in the food industry. The research result may directly contribute to the value of these commodities for use in various food applications, and developing new food and feed products from them, therefore supporting expanding production of climate-resilient legumes, such as Vigna species, in the diversified farming systems of the Australian tropics.

## 2. Materials and Methods

### 2.1. Materials

Moth bean grains of 3 different cultivars (AVTMB#MK, AVTMB#PB, and AVTMB#BM) and cowpea grains of 3 different cultivars (AVTCP#5, CPTX-03, and CPTX-08), grown in the Australian subtropical climate of central Queensland in 2023, were provided by the Institute for Future Farming Systems (IFFS) of CQUniversity (Rockhampton, Australia).

### 2.2. Starch Isolation

The starch was isolated as a byproduct of protein isolation, followed by the method of Kumar et al. (2025) [18]. Briefly, 100 g of each legume grain was soaked in distilled water (1:10 *w*/*v*) overnight at room temperature to facilitate dehulling. The hydrated grains were manually dehulled and homogenised with water (1:10 *w*/*w*), using a blender to obtain a uniform slurry. The slurry was adjusted to pH 9.0, using 0.2 M NaOH, and stirred at 900 rpm for 1 h at 20 °C to solubilise the protein. After standing for 1 h to allow for starch sedimentation, the supernatant was decanted, and the starch-rich sediment was collected. The sediment was repeatedly washed with distilled water (three times, 1:5 *w*/*v*) and centrifuged at 10,000× *g* for 5 min to remove residual protein and fibre. The collected starch was neutralised to pH 6.5–7.0 with 0.2 M HCl, then washed twice with 95% ethanol to eliminate the pigments and lipid impurities. The purified starch was dried in a convection oven at 40 °C for 12 h, ground into fine powder, and passed through a 100-mesh sieve. The dried starch samples were stored in a desiccator at room temperature until further analyses.

### 2.3. Microscopy

The starch granule morphology was observed using a light microscope (DMi8, Leica, Wetzlar Germany), equipped with a camera. Starch samples were suspended in Milli-Q water, mounted on microscope slides, and covered with cover slips. The morphology was examined at 20× magnification.

### 2.4. Particle Size Distribution

Particle size distribution of samples was determined using a Malvern Mastersizer 2000 (Malvern Instruments, Worcestershire, UK) [19]. The sample was suspended in MilliQ water and mixed to disperse the agglomerate before measurement. The obscuration was kept in the range of 13–15%, and 1.33 was used as the refractive index for size measurement.

### 2.5. Apparent Amylose Content (ACC)

The ACC of starch samples was measured by the iodine colorimetric method described by Hoover et al. (2001) [20], with some modifications. Briefly, the samples (20 ± 0.1 mg) were dispersed in 8 mL of 90% DMSO, vortexed for 1 min, and treated as per amylose/amylopectin calibration mixtures prepared from potato amylose (A-0512, Sigma-Aldrich, St. Louis, MO, USA) and maize amylopectin (S-9765, Sigma-Aldrich, St. Louis, MO, USA). The samples were heated in an 85 °C water bath for 15 min with intermittent mixing, then cooled, and diluted to 25 mL. An aliquot (0.1 mL) was mixed with 4.4 mL distilled–deionised water and 0.5 mL iodine reagent (0.0025 M I_2_/0.0065 M KI) in a 10 mL volumetric flask. After vertexing and 15 min incubation at room temperature, absorbance was recorded at 640 nm, and the amylose content was calculated from the calibration curve.

### 2.6. X-Ray Diffraction (XRD)

XRD patterns of starch samples were obtained using an X-ray diffractometer (Miniflex 600, Rigaku Corporation, Tokyo, Japan) with Cu Kα radiation (40 kV, 20 mA) [21]. Scans were performed from 5° to 30° (2θ), with a 0.02° step size at 1°/min. Relative crystallinity was calculated as the ratio of the crystalline peak area to the total diffraction area, using the PeakFit software (Version 4.0, Systat Software Inc., San Jose, CA, USA).

### 2.7. Gelatinisation and Retrogradation

The starch gelatinisation properties were measured with a differential scanning calorimeter (DSC 2500, TA Instruments, New Castle, DE, USA), based on a previous method [22] with slight modifications. The starch sample (5.0 mg, dry-weight basis) was weighed into a zero-aluminium liquid DSC pan, mixed with 10 μL distilled water, and hermetically sealed. The samples were equilibrated for 1 h at room temperature, and scanned from 20 to 110 °C at 5 °C/min. An empty sealed pan served as a reference. Onset (*T_o_*), peak (*T_p_*), and conclusion (*T_c_*) temperatures, along with enthalpy change (Δ*H*), were obtained using TA Universal Analysis software (version 5).

For determining retrogradation properties, scanned pans were stored at 4 °C for 7 days and rescanned under the same condition. The onset (*T_o_*(r)), peak (*T_p_*(r)), and conclusion (*T_c_*(r)) temperatures and enthalpy of retrograded starch (Δ*H*(r)) were determined, and retrogradation percentages (R%) were calculated as the following:

R% = ΔH(r)/ΔH × 100

### 2.8. Pasting Properties

The pasting properties were determined with a Rapid Visco Analyser (RVA 4800, PerkinElmer, Waltham, MA, USA). A total of 3 g of the starch sample was mixed with 25 mL distilled water in an RVA canister. The temperature profile began at 50 °C for 1 min, increased linearly to 140 °C for 7 min and held for 7 min, cooled linearly to 50 °C for 7 min, and held for an additional 2 min. Mixing speed was at 960 rpm for the first 10 s, then reduced to 160 rpm for the remaining 20 min of the test.

### 2.9. Texture Analysis

The legume starch gel formed in the canister during RVA analysis was sealed with Parafilm and placed in a refrigerator at 4 °C for 24 h. Gel strength and the rupture strength of starch gels were measured with a TAXT plusC texture analyser (Stable Micro Systems Ltd., Surrey, UK), fitted with a 10 mm cylindrical probe (P/10). Gels were compressed to 10 mm depth at 2 mm/s. Gel strength was calculated as the peak force during the first compression cycle, and rupture strength was calculated as the maximum force recorded at the point of gel break during the puncture test.

### 2.10. Cluster Heatmap Analysis

To investigate the relationships among starch physicochemical parameters and to visualise grouping patterns across different starch samples, hierarchical cluster analysis was performed using OriginPro 2025. Pearson’s correlation coefficient was applied to group starch samples as the similarity metric and average linkage method, based on their physicochemical parameters.

### 2.11. Statistical Analysis

All experimental data were expressed as means ± standard deviations from triplicate measurements. Each of the six cultivars (three cowpea and three moth bean) was treated as an independent sample and analysed individually. Statistical comparisons among all six samples were conducted using one-way analysis of variance (ANOVA), followed by Tukey’s multiple-range test to identify significant differences (*p* < 0.05). Superscript letters in tables indicate statistically significant differences among cultivars. All statistical analyses were performed using SPSS 26.0 software, and all figures were prepared using Microsoft Office Excel.

## 3. Results and Discussion

### 3.1. Morphology and Particle Size of Starch

**Figure 1:**
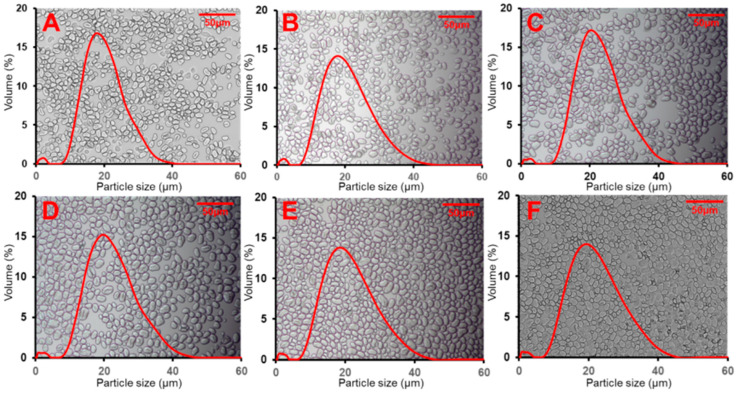
Light microscopic pictures and size distribution of starch samples (**A**) AVTMB#MK; (**B**) AVTMB#PB; (**C**) AVTMB#BM; (**D**) AVTCP#5; (**E**) CPTX-03; and (**F**) CPTX08.

Representative light microscopic images of the isolated starches are shown in Figure 1. All six legume cultivars displayed starch granules with consistent oval to spherical shapes, and no discernible morphological differences were observed among the cultivars under light microscopy, suggesting a relatively uniform granule morphology across all samples. These observations are consistent with previous reports on moth bean [10] and cowpea starches [23].

The particle size distribution curves (Figure 1) of all starch samples were unimodal, with a single dominant peak appearing between approximately 15 and 25 μm, indicating that the starch granules exhibited a relatively homogeneous size distribution. Quantitative measurements of granule size (*D* [4, 3]), which represent the average particle size, are summarised in Table 1, and range from 15.86 μm to 18.35 μm. Significant differences were observed among the cultivars. Within the moth bean starches, AVTMB#BM exhibited the largest mean diameter (18.35 ± 0.01 μm), while AVTMB#PB showed the smallest granules (15.86 ± 0.14 μm). In the cowpea group, AVTCP#5 had the largest particle size (17.54 ± 0.02 μm), followed by CP-TX08 (17.16 ± 0.03 μm) and CP-TX03 (16.52 ± 0.02 μm). These results indicate that both inter-species (moth bean vs. cowpea) and intra-species (genotypic) variation exists in starch granule size under identical growing conditions. This is consistent with previous reports on legume starches, which have shown that granule size is largely influenced by cultivar and species-specific characteristics [16,24]. The observed differences in granule size may further impact starch’s functional properties, such as thermal and gelatinisation characteristics, crystallisation level, and starch gel texture.

### 3.2. Apparent Amylose Content (AAC) and Crystallinity

Quantitative analysis of AAC revealed a broad variation among the six legume starches, ranging from 22.2% to 33.9% (Table 1). In general, cowpea starches exhibited higher AAC values compared to moth bean starches. Among them, the AVTCP#5 showed the highest AAC (33.9 ± 1.4%), followed by CP-TX08 (32.23 ± 1.3%) and CP-TX03 (28.1 ± 1.4%). In contrast, the three moth bean starches (AVTMB#MK, AVTMB#BM, and AVTMB#PB) showed relatively lower AAC values (from 27.4% to 22.2%). These variations reflect both species-level and cultivar-level diversity, which are likely influenced by the genetic background and biosynthetic regulation of starch structure.

**Figure 2:**
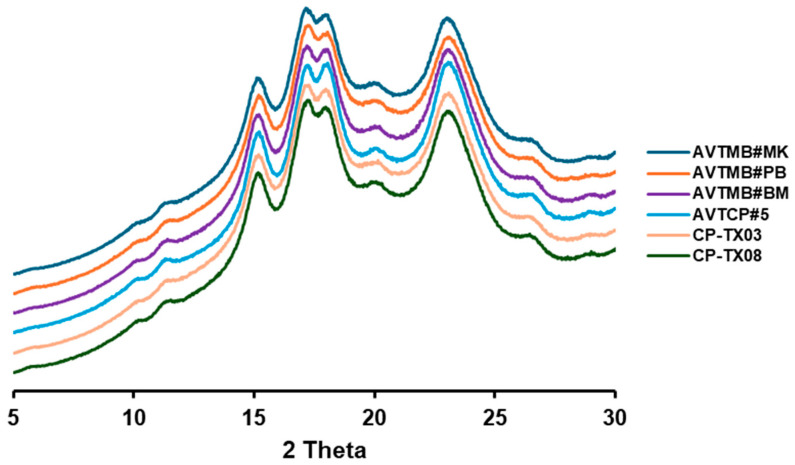
X-ray diffractograms of isolated starch samples.

The crystalline structure of starch granules is commonly characterised by using X-ray diffraction (XRD). The X-ray diffractograms of six legume starch samples are presented in Figure 2. All starch samples exhibited typical C-type X-ray diffraction patterns, characterised by prominent peaks at 15°, 17°, 18°, and 23° (A-type contribution), along with weaker peaks or shoulder peaks around 5.6° and 20° (B-type contribution), consistent with previous reports on legume starches [25,26]. The relative crystallinity (RC) of starch ranged narrowly between 33.43% and 35.68%. However, we still can find that compared with the cowpea starches, the moth bean starches have a relative higher RC. Notably, AVTMB#PB, which had the lowest AAC, exhibited the highest RC (35.68%), whereas AVTCP#5, with the highest AAC, showed the lowest RC (33.43%). This result is consistent with previous research, which reported that the starch with higher AAC usually exhibit lower RC [16,27,28], because the amylopectin is primarily responsible for forming crystalline regions through its ability to pack into ordered double helices, while amylose is mostly linear, tends to disrupt this packing, and contributes to the amorphous regions [29].

### 3.3. Thermal and Retrogradation Properties

**Figure 3:**
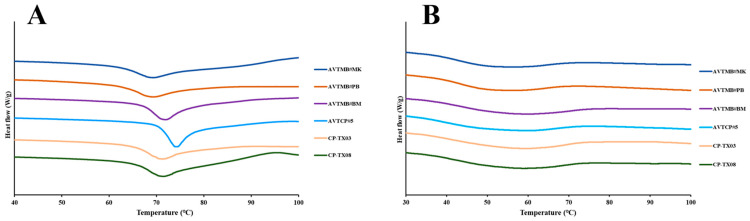
Thermograms of cowpea and moth bean starch samples. ((**A**) raw starch samples; (**B**) retrograded starch samples).

The thermal properties of moth bean and cowpea starches (*T_o_*, *T_p_*, *T_c_* and Δ*H*) are presented in Table 2, and the corresponding thermograms are presented in Figure 3. All starch samples exhibited typical gelatinisation endotherms within the range reported for moth bean and cowpea starches [30,31], with the *T_o_* ranging from 61.27 to 70.43 °C, *T_p_* from 69.21 to 74.15 °C, and *T_c_* from 93.40 to 94.83 °C. The gelatinised temperatures were generally higher in cowpea starches, particularly for AVTCP#5, which had the highest amylose content, suggesting that a higher proportion of amylose contributes to greater thermal stability of starch granules, as amylose tends to form more rigid and less readily hydrated structures than amylopectin, thereby requiring more energy and higher temperatures to disrupt the crystalline structure during gelatinisation [32,33]. The gelatinisation enthalpy (Δ*H*) represents the energy required to unwind double helices and melt the crystalline domains that are predominantly formed by amylopectin [34]. In our study, the moth bean starch samples, which have a higher amylopectin content, generally exhibited higher Δ*H* values compared with cowpea starches, indicating a more extensive crystalline organisation in moth bean starches, likely due to a greater abundance of amylopectin double helices contributing to ordered lamellar structures [35]. Consequently, more thermal energy was required to disrupt these ordered regions during gelatinisation.

After 7 days of refrigerated storage, all starch samples displayed clear retrogradation endotherms (Table 2 and Figure 3B). The gelatinisation temperatures after retrogradation were generally higher in cowpea starches, compared with moth bean starches, which was consistent with previous studies, indicating that starches with a higher amylose content tend to exhibit more extensive retrogradation and form thermally stable recrystallised structures [36,37,38]. This trend suggests that amylose chains in cowpea starches reassociate more effectively during cold storage, leading to the formation of well-ordered double helices that require more energy to dissociate upon reheating.

The percentage of retrogradation (R%) was calculated as the ratio of retrograded enthalpy (Δ*H*(r)) to the original gelatinisation enthalpy (Δ*H*). The R% values varied considerably among the samples, ranging from 48.12% to 75.73%, reflecting notable differences in the extent of starch chain reassociation during refrigerated storage. Notably, the cowpea starches exhibited significantly higher R% values (64.17–75.73%) compared to the moth bean starches (48.12–50.12%). This result is consistent with the higher amylose content in cowpea starch samples, as amylose is more prone to reassociation into a double helix, and undergoes retrogradation during cold storage [36,38].

### 3.4. Pasting Properties

Further assessment of the functional implications of starch structure and composition pasting behaviours were analysed using RVA. The pasting properties of the six starch samples are presented in Table 3. Overall, the viscosity parameters, including peak viscosity, breakdown, final viscosity, and setback were comparable among the samples, suggesting that the swelling, disintegration, and realignment of starch granules during heating and cooling occurred to a similar extent across both legume species and their cultivars.

In contrast, pasting temperature, which reflects the onset of starch granule swelling and partial gelatinisation under combined shear and thermal conditions [39], exhibited more pronounced variation. Notably, cowpea starches displayed higher pasting temperatures (up to 80 °C), compared with moth bean starches. This trend aligns with the higher amylose content and gelatinisation temperatures observed in DSC measurements for cowpea samples.

The increased pasting temperature suggests that the more rigid granular architecture and reduced hydration capacity associated with higher amylose content confer greater thermal resistance, thereby delaying the onset of pasting [40,41].

### 3.5. Texture Properties

**Figure 4:**
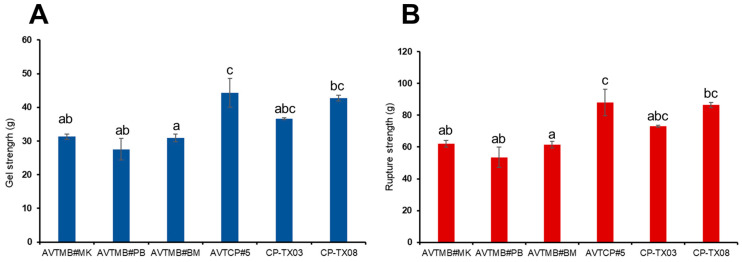
Texture profile analysis of isolated starch samples. (**A**) Gel strength (g); (**B**) rupture strength (g). Bars with different lowercase letters indicate significant differences among samples (*p* < 0.05).

For the mechanical integrity and consumer-relevant characteristics of starch-based gels, texture measurements were performed using a texture analyser. The texture profile analysis of starch gels, including hardness, rupture strength, and adhesiveness, is summarised in Figure 4. Significant variations in hardness were observed among the samples (Figure 4A), ranging from 27.52 g (AVTMB#PB) to 44.34 g (AVTCP#5). Notably, cowpea starches exhibited significantly higher gel hardness than moth bean starches. This increased hardness is primarily attributed to starch retrogradation, particularly the recrystallisation of amylose, which promotes the formation of firmer gel networks [42,43].

This observation is consistent with the higher apparent amylose content found in cowpea starches, as amylose plays a crucial role in strengthening the gel matrix, due to its linear structure, which facilitates intermolecular associations, and the development of ordered, rigid networks upon cooling. Additionally, the greater retrogradation tendency (as indicated by higher R% values) observed in cowpea starches likely further reinforces their gel structure, resulting in firmer textures, compared to their moth bean counterparts. A similar trend was observed for rupture strength (Figure 4B), which is defined as the force required to break the gel matrix, and reflects the gel’s internal cohesion [44]. Cowpea starch gels demonstrated greater resistance to structural failure, indicating the formation of more compact and mechanically stable gel networks.

Overall, the texture profile analysis indicates that compared with moth beans, cowpea starches can form more rigid and mechanically stable gels, reflecting their compositional and structural advantages for applications requiring a firm texture.

### 3.6. Cluster Heatmap Analysis

**Figure 5:**
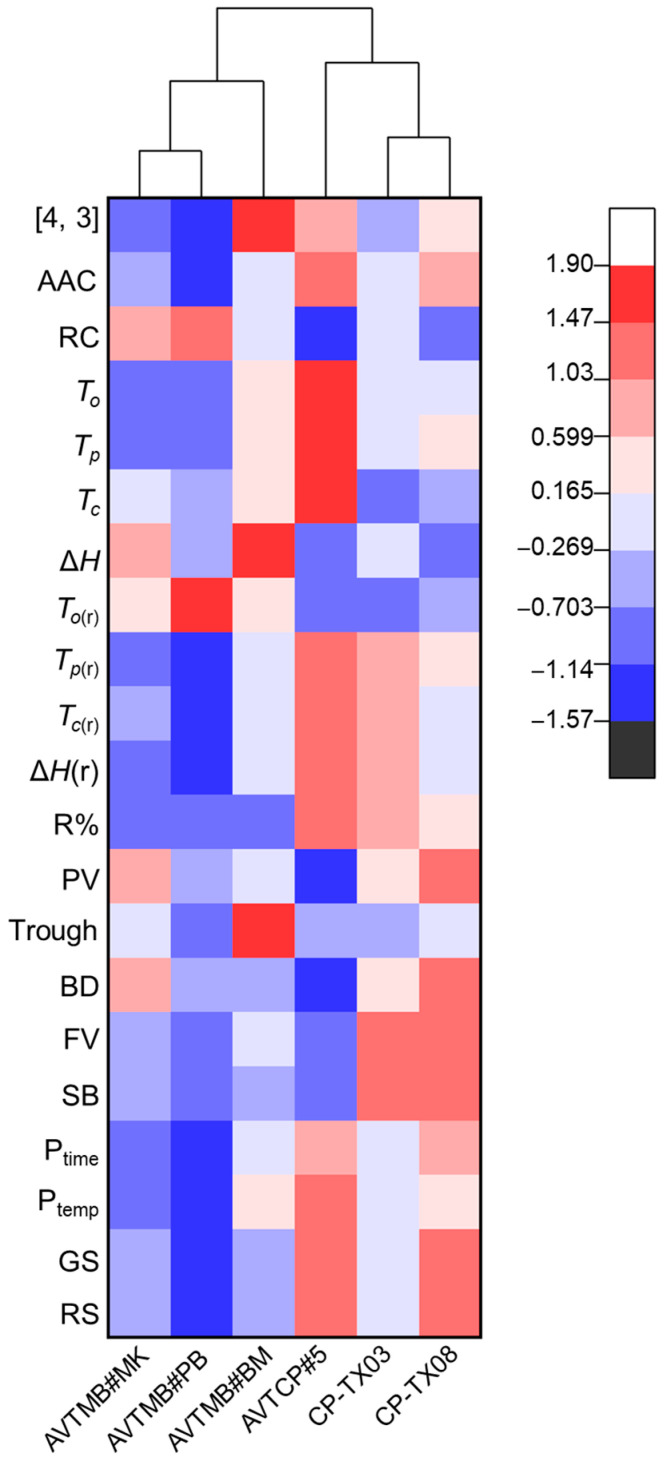
Hierarchical clustering heatmap of physicochemical and functional properties of isolated starch samples. Abbreviations represent starch physicochemical parameters: *D* [4, 3] (volume-weighted mean diameter), AAC (apparent amylose content), RC (relative crystallinity), *T_o_* (onset gelatinisation temperature), *T_p_* (peak gelatinisation temperature), *Tc* (conclusion gelatinisation temperature), Δ*H* (gelatinisation enthalpy), T*_o_*_(r)_ (onset gelatinisation temperature), T*_p_*_(r)_ (peak gelatinisation temperature), T*_c_*_(r)_ (conclusion gelatinisation temperature), Δ*H*_(r)_ (gelatinisation enthalpy), R% (percentage of retrogradation), PV (peak viscosity), Trough (Through viscosity), BD (breakdown viscosity), FV (Finial viscosity), SB (setback viscosity), P_time_ (peak time), P_temp_ (peak temperature), GS (gel strength), RS (rupture strength).

To further evaluate the multivariate relationships among starch samples, a hierarchical clustering heatmap was generated, using Pearson correlation as the distance metric (Figure 5). The dendrogram revealed two distinct clusters that aligned with the botanical origin of the starch samples. One cluster comprised the three moth bean starches (AVTMB#MK, AVTMB#PB, AVTMB#BM), while the other included the three cowpea starches (AVTCP#5, CP-TX03, CP-TX08). This separation primarily reflects differences in structural and physicochemical properties in starch between the two legume species, particularly in relation to amylose content, thermal and retrogradation properties, pasting behaviours, and gel textural characteristics.

Samples within the cowpea cluster were characterised by higher apparent amylose content, gelatinisation temperatures, and retrogradation enthalpy. These features indicate an enhanced thermal stability and retrogradation tendency, primarily due to the ability of amylose chains to reassociate and form dense, amorphous networks during storage [32,33,36,38].

In terms of functional behaviour, cowpea starches also exhibited higher pasting temperatures, gel strength, and rupture strength, all of which further underscore their greater structural rigidity and stronger intermolecular interactions [42,43]. These trends suggest that amylose-rich cowpea starches undergo more extensive retrogradation and form mechanically robust gels during cold storage, highlighting their potential utility in food formulations requiring firm and stable gel structures.

Conversely, the moth bean starches formed a distinct cluster, marked by lower apparent amylose content, gelatinisation temperatures, and gel strength, but higher relative crystallinity and gelatinisation enthalpy. These observations suggest a higher degree of crystalline organisation in the native starch granules. However, the reduced amylose content in moth bean samples limits their ability to undergo extensive chain reassociation during storage, resulting in diminished retrogradation behaviour [28]. Consequently, starch gels derived from moth beans exhibited softer textures and lower mechanical resilience compared to cowpea starches. Such characteristics may render moth bean starches more suitable for applications in food systems that require soft and smooth gel textures with a low retrogradation tendency.

## 4. Conclusions

This study provided a comparative analysis of structural and functional characteristics of starches isolated from three cowpea and three moth bean cultivars. Notable differences were observed between the two legume species in terms of amylose content, crystallinity, gelatinisation and retrogradation behaviour, pasting properties, and gel texture. Cowpea starches were characterised by higher amylose content, greater thermal stability, stronger retrogradation tendency, higher pasting temperatures, and firmer gel textures. In contrast, moth bean starches displayed lower amylose content, higher relative crystallinity, and lower retrogradation tendency, resulting in softer gels with limited structural reinforcement during cold storage.

Despite relatively minor variation among cultivars within each species, the clear clustering pattern revealed by clustering heatmap analysis confirmed that the botanical origin plays a dominant role in determining starch behaviour. The distinct functional profiles of cowpea and moth bean starches suggest different suitability for food applications. Cowpea starches are more appropriate for uses requiring thermal and structural robustness, such as noodle formulations and microwave foods, whereas moth bean starches may be better suited for applications needing smooth textures and minimal retrogradation, such as in instant or ready-to-eat food products.

These findings expand our understanding of underutilised legume starches and provide valuable insights for their potential use in tailored food product development, supporting the broader utilisation of alternative starch sources in sustainable food systems. Future studies should further investigate their digestibility and functionality in complex food matrices to strengthen their potential for broader industrial use.

## Figures and Tables

**Table 1:** Particle size (D), apparent amylose content (AAC), and relative crystallinity (RC) of isolated starch samples ^1^.

Samples	*D* [4, 3] (μm) ^2^	AAC (%)	RC (%)
AVTMB#MK	16.17 ± 0.01 ^b^	27.40 ± 0.71 ^b^	35.27 ± 0.32 ^ab^
AVTMB#PB	15.86 ± 0.14 ^a^	22.23 ± 1.65 ^a^	35.53 ± 0.12 ^b^
AVTMB#BM	18.35 ± 0.01 ^f^	27.57 ± 1.70 ^bc^	34.68 ± 0.24 ^ab^
AVTCP#5	17.54 ± 0.02 ^e^	33.90 ± 1.47 ^d^	33.75 ± 0.41 ^a^
CP-TX03	16.52 ± 0.02 ^c^	28.07 ± 1.43 ^bc^	34.49 ± 0.21 ^ab^
CP-TX08	17.16 ± 0.03 ^d^	32.23 ± 1.31 ^cd^	33.93 ± 0.32 ^a^

^1^ Values are means ± SD. Values in the same column with the same letters do not differ significantly (*p* < 0.05); ^2^ *D* [4, 3], AAC, and RC represent weighted mean value by volume, apparent amylose content, and relative crystallinity, respectively.

**Table 2:** Thermal and retrogradation parameters of cowpea and moth bean starch samples ^1^.

Samples	*T_o_* (°C) ^2^	*T_p_* (°C)	*T_c_* (°C)	Δ*H* (J/g)	*T_o_*(r) (°C)	*T_p_*(r) (°C)	*T_c_*(r) (°C)	Δ*H*(r) (J/g)	R%
AVTMB#MK	61.27 ± 0.14 ^a^	69.21 ± 0.08 ^a^	93.78 ± 0.22 ^a^	17.43 ± 0.35 ^bc^	37.11 ± 0.12 ^ab^	51.90 ± 0.18 ^a^	72.19 ± 0.52 ^ab^	8.46 ± 0.29 ^ab^	48.56
AVTMB#PB	61.88 ± 0.19 ^a^	69.21 ± 0.08 ^a^	93.40 ± 0.47 ^a^	15.31 ± 0.47 ^ab^	37.81 ± 0.19 ^b^	51.56 ± 0.59 ^a^	69.19 ± 1.46 ^a^	7.67 ± 0.35 ^a^	50.12
AVTMB#BM	66.22 ± 0.12 ^c^	72.07 ± 0.03 ^c^	93.92 ± 1.05 ^a^	18.88 ± 0.36 ^c^	36.90 ± 0.43 ^ab^	54.74 ± 1.79 ^ab^	74.13 ± 1.06 ^bc^	9.09 ± 0.13 ^abc^	48.12
AVTCP#5	70.43 ± 0.08 ^d^	74.15 ± 0.10 ^d^	94.83 ± 1.53 ^a^	15.05 ± 0.45 ^a^	36.11 ± 0.49 ^a^	58.22 ± 0.76 ^b^	79.00 ± 1.63 ^d^	11.34 ± 1.61 ^c^	75.37
CP-TX03	64.79 ± 0.25 ^b^	71.35 ± 0.20 ^b^	93.01 ± 1.65 ^a^	15.85 ± 1.08 ^ab^	36.22 ± 0.78 ^a^	57.13 ± 1.07 ^b^	77.24 ± 1.89 ^c^	10.59 ± 0.68 ^bc^	66.83
CP-TX08	64.60 ± 0.51 ^b^	71.72 ± 0.16 ^bc^	93.38 ± 1.03 ^a^	14.72 ± 0.44 ^a^	36.60 ± 0.04 ^a^	56.49 ± 0.09 ^b^	73.81 ± 0.24 ^abc^	9.44 ± 0.12 ^abc^	64.17

^1^ Values are means ± SD. Values in the same column with the same letters do not differ significantly (*p* < 0.05); ^2^ *T_o_*, *T_p_*, *T_c_*, and Δ*H* are onset, peak, and conclusion temperatures, and enthalpy change in gelatinisation, respectively; *T_o_*(r), *T_p_*(r), *T_c_*(r), and Δ*H*(r) are onset, peak, and conclusion temperatures, and enthalpy change in retrogradation, respectively.

**Table 3:** Pasting properties of cowpea and moth bean starch samples ^1^.

Samples	Peak Viscosity (mPa·s)	Trough (mPa⋅s)	Breakdown (mPa⋅s)	Final Viscosity (mPa⋅s)	Setback (mPa⋅s)	Peak Time (min)	Pasting Temperature (°C)
AVTMB#MK	5559.5 ± 112.5 ^ab^	871.5 ± 6.5 ^a^	4688.0 ± 119.0 ^b^	2434.5 ± 36.5 ^a^	1563.0 ± 30.0 ^a^	4.5 ± 0.1 ^ab^	76.0 ± 0.4 ^a^
AVTMB#PB	5223.5 ± 163.5 ^ab^	843.5 ± 17.5 ^a^	4380.0 ± 146.0 ^ab^	2380.0 ± 65.0 ^a^	1536.5 ± 47.5 ^a^	4.4 ± 0.0 ^a^	74.8 ± 0.0 ^a^
AVTMB#BM	5321.5 ± 44.5 ^ab^	929.0 ± 5.0 ^a^	4392.5 ± 49.5 ^ab^	2488.0 ± 43.0 ^a^	1559.0 ± 38.0 ^a^	4.6 ± 0.0 ^bc^	78.0 ± 0.1 ^b^
AVTCP#5	5060.5 ± 61.5 ^a^	850.0 ± 6.0 ^a^	4210.5 ± 55.5 ^a^	2388.5 ± 15.5 ^a^	1538.5 ± 21.5 ^a^	4.7 ± 0.0 ^c^	80.0 ± 0.5 ^c^
CP-TX03	5446.0 ± 50.0 ^ab^	847.0 ± 39.0 ^a^	4599.0 ± 11.0 ^ab^	2592.5 ± 47.5 ^a^	1745.5 ± 8.5 ^a^	4.6 ± 0.0 ^bc^	77.6 ± 0.4 ^b^
CP-TX08	5597.0 ± 28.0 ^b^	869.5 ± 23.5 ^a^	4727.5 ± 4.5 ^b^	2588.0 ± 66.0 ^a^	1718.5 ± 89.5 ^a^	4.7 ± 0.0 ^c^	78.0 ± 0.0 ^b^

^1^ Values are means ± SD. Values in the same column with the same letters do not differ significantly (*p* < 0.05).

## Data Availability

The original contributions presented in this study are included in the article. Further inquiries can be directed to the corresponding author.
